# Domestic

**Published:** 1841-08-21

**Authors:** 


### DOMESTIC.

The last number of the Western Journal of Medicine and Surgery contains a case of diaphragmatic hernia, with the dissection by Dr. Bayless. The rarity of the case, and the accuracy of the dissection, induces us to extract its main features. The patient, a German, æt. 35, was variously deformed, the head was very large, the thighs slightly but permanently flexed upon the trunk, while the legs were flexed almost at right angles upon the thighs, owing, as was supposed, to a congenital contraction of the flexor muscles of the thigh, he having been so from birth.

His sister, who accompanied him at the time of his admittance, stated that he had been idiotic, had curved spine, and flexed lower extremities, from birth—that he had never walked, and it had always been necessary to carry him about; but that he had enjoyed rather robust health. From the time of his admittance, he remained altogether in his bed, sometimes in the sitting, but mostly in the recumbent posture—was observed to be an enormous eater—that his bowels were habitually constipated, there being usually an interval of from eight to fourteen days between the time of his having an evacuation, which always consisted of an immense bulk of hardened fecal matter—but that his general health remained good, having only an occasional attack of dyspnœa, which was slight in its character, of from half an hour to three-quarters in duration, and brought on by mental excitement, such as being irritated by his fellow inmates, but more frequently by rising suddenly in bed. The attention of the visiting physician was never called to him, and the resident students never made any examination in reference to the action of the heart. He never complained of pain in the thorax or abdomen, until he was attacked with dysentery, when there was only the pain that is usually attendant upon that disease, which was prevailing in the house at the time, and of which he died.

The examination was made twenty-nine hours after death. Upon laying open the abdomen to examine the bowels in reference to the disease of which he died, the stomach was observed to be out of its usual place; or rather, there was only a small portion of its pyloric extremity visible. Tracing it from this, we found it empty, and that almost the whole organ, which was of maximum size, had passed through a large opening in the diaphragm into the cavity of the thorax. The opening consisted in an enlargement of the natural foramen for the transmission of the œsophagus, was circular in shape, fully two inches and a half in diameter, with its edges about a third of an inch thick. The peritoneum, as it covers the diaphragm, was reflected through the opening around its whole edge, passed vertically about four inches into the cavity of the thorax, thus forming a hollow cylinder of that length, and two inches and a half in diameter; and having reached that height, the membrane concentrated upon the œsophagus, which was placed in the axis of the cylinder, and thence passed down it upon the stomach, and on to the other viscera. Thus was formed a *cul de sac*, in which the stomach was lying; but we should remark, however, that it was not perfectly cylindrical, for that it was enlarged above so as to make it somewhat balloon shaped. The peritoneum forming the sac, was thickened to about double of what it usually is. We found no lesion of any of the viscera of the abdomen, having any connexion with that of the diaphragm. Upon opening the thorax, the left cavity was observed to be somewhat smaller than the right, in consequence of the protruding stomach, which was found lying in the posterior mediastinum, trespassing slightly upon this cavity; but, notwithstanding, it was sufficiently capacious to allow the natural function of the lung, which was in a normal condition. The right lung was adherent throughout almost its whole surface to the contiguous parts by old adhesions, and so firmly to the diaphragm as to require a very careful dissection to effect its separation. The stomach, (as has been observed,) with its containing peritoneal *cul de sac*, was lying in the posterior mediastinum, in the natural position of the œsophagus, inclining therefore to the left side of the spine, and infringing more upon the left than the right pleural sac. There was no perforation of either of the pleuræ; and at the base of the protruding peritoneal sac, for near an inch above the edge of the diaphragm, the left pleura, as it commences ascending to form the mediastinum, was separated from the peritoneum by only a thin layer of cellular substance; but above this a greater amount of loose cellular texture was interposed between them. The protruding parts were not found to pass sufficiently high and to the left to offer any mechanical interference with the action of the heart, which was in a normal condition; but it will be recollected that we could gather no facts relative to the manner in which this organ discharged its duty.

Whether as mere coincidences, or as having a necessary connexion with those already described, we pretend not to say, but as occurring in like structures, your committee deem it right to mention briefly some lesions which were found in the head; and the condition of the spine to which the curvature was owing. In the first place, the bones of the head generally, but particularly those forming the vault of the cranium, were in a state of manifest hypertrophy, being in some places well nigh half an inch thick. On their inner surface, (especially the parietal and frontal bones,) were found a considerable number of exostoses, in the form of conical projections, about the fourth of an inch high, and the third of an inch in diameter at the base. The dura-mater adhered with unusual firmness to the skull, but particularly to these projections. On the inner surface of the dura-mater were found a number of osseous deposits. These existed most abundantly in the falx cerebri, along the superior longitudinal sinus; where they varied in size from masses of an oblong shape, an inch long and half an inch wide, to mere points. There were some of them scattered throughout the whole of the falx major and tentorium; and wherever found consisted of very hard, white osseous masses, very rough on their free or cerebral surface, but smooth on that by which they adhered to the dura mater. The distortion of the spine consisted in a posterior curvature, which was somewhat gradual between the first dorsal and last lumbar vertebræ, being greatest, however, at the middle of the dorsal, where the deviation from the natural position was about two inches and a half. There was also an observable curvature to the right side. The bony structure of the column seemed to be in a perfectly healthy condition; but the intervertebral substance, in all the spaces included within the curvature, was deficient in its anterior part, and from the first to the eighth dorsal vertebra, the absorption was so complete as to bring the anterior edges of the vertebræ immediately into contact. This absorption or atrophy of the intervertebral substance, may have happened in one of two ways; either as an original or congenital malformation, or by atrophy of the erector muscles of the back, by which the parts in front of the spine would preponderate and cause the anterior portion of the intervertebral substance to be unduly pressed, and absorption results as a consequence. The former we think most probably true; as it was stated, by his sister, that the curvature existed from birth.

---

We quote the two following cases as corroborative of a point of considerable practical importance, to which we have already directed attention on one or two occasions, viz.: the ease with which hæmorrhage, from a wounded radial or ulnar artery, may frequently be arrested by simple compression, and as perfect and permanent a cure effected as could be obtained from the employment of a ligature. It would even appear, from the first of the subjoined cases, that hæmorrhage may possibly be arrested without the necessary obliteration of the wounded vessel. The point of interest, however, is the fact that a hæmorrhage, as profuse as that from a divided radial or ulnar artery can be effectually arrested by compression; and that a practitioner in the country, who should have paid but little attention to surgery, and who would be embarrassed in his attempts to take up the divided vessel, has presented to him a safe and simple treatment in the application of a well graduated compression.

*Cases of Wounded Arteries of the Arm, relieved by the Bandage:* Reported to the Medical Society of Tennessee, May, 1841. By Dr. GEORGE THOMPSON, of Jefferson County, Tennessee.

CASE I.—A young man received a wound from a long knife in the fore-arm. The knife entered about the middle of the fore-arm, and passing obliquely upward, wounded the ulnar artery just below the point of separation from the radial. The hæmorrhage had been considerable, but was arrested by the application of a bandage around the arm above the elbow. In this situation he came to my shop, an hour after he had received the injury. A compress was laid over the course of the wound, and another over the brachial artery, at the point where that vessel could be most conveniently compressed against the humerus. A roller was then carefully applied from the points of the fingers to the shoulder, so tightly as barely to permit a sufficient quantity of circulation to maintain the vitality of the limb. Cold water was freely applied to the whole arm; he was put on light diet, with an occasional dose of epsom salts. I removed and re-adjusted the bandage daily, to satisfy myself that the limb was receiving no injury from it. In ten or twelve days the wound was healed. On removing the bandage the circulation was found to be carried on through the wounded vessel near the wrist as freely as before the wound was received. I could not satisfy myself that the canal of the vessel was not obliterated at the wounded point, from the depth with which it was covered by the integuments; but I am of the opinion it was not.

CASE II.—A young man passed a sharp pointed narrow knife under the tendon of the extensor muscle of the thumb, entering at the point where the radial artery passes under that tendon, wounding that vessel and passing out at the opposite side of the wrist. A compress was applied along the course of the wound, another on the vessel above the wound, a bandage was firmly applied to the hand and fore-arm, and the patient was left with instruction to let me know if the bandage produced much pain. His hand becoming painful in the night, he had the bandage taken off and applied loosely. I heard nothing more from him for four or five days, when he came to me to examine his hand. On taking off the bandage, which was quite loose, I found the wounds in the skin healed, and a strongly pulsating tumor along the whole course of the wound. The compresses and bandage were again applied as at first. The bandage was now permitted to remain as I applied it, and in three weeks all traces of aneurism had disappeared, and the hand was soon restored to its original condition.

### HEALTH OF THE CITY

INTERMENTS *in the City and Liberties of Philadelphia, from the 7th to the 14th of August.*

| Diseases. | Adults. | Children. | Diseases. | Adults. | Children. |
|---|---|---|---|---|---|
| Abscess, | 0 | 1 | *Brought forward,* | 41 | 52 |
| Croup, | 0 | 3 | Neglect, | 0 | 2 |
| Cholera Morbus, | 1 | 1 | Old age, | 2 | 0 |
| Colic, | 1 | 0 | Palsy, | 2 | 0 |
| Consumption of the lungs, | 11 | 3 | Pleurisy, | 1 | 0 |
| Convulsions, | 0 | 8 | Scirrhus, | 1 | 0 |
| Diarrhœa, | 1 | 10 | Scrofula, | 0 | 2 |
| Dropsy, | 2 | 1 | Small pox, | 2 | 1 |
| Dropsy of the head, | 0 | 4 | Still-born, | 0 | 6 |
| Disease of Hip, | 0 | 1 | Summer Complaint, | 0 | 27 |
| Drowned, | 3 | 2 | Syphillis, | 1 | 0 |
| Dysentery, | 5 | 4 | Teething, | 0 | 2 |
| Debility | 1 | 3 | Tabes Mesenterica, | 0 | 1 |
| Erysipelas, | 0 | 1 | Ulcers, | 1 | 0 |
| Enlargement of Liver, | 1 | 0 | Unknown, | 1 | 1 |
| Fever, | 1 | 0 | Vomiting of Blood, | 0 | 1 |
| —— Bilious, | 1 | 0 | | — | — |
| —— Typhus, | 2 | 0 | Total, | 156—51 | 105 |
| —— Typhoid, | 1 | 0 | | | |
| Inflammation of the Brain, | 2 | 4 | | | |
| —— Breast, | 0 | 1 | | | |
| —— Lungs, | 3 | 0 | | | |
| —— Stomach and Bowels, | 0 | 2 | | | |
| —— Bowels, | 2 | 3 | | | |
| Inflammation of the Heart, | 0 | 1 | | | |
| —— Peritonæum, | 1 | 0 | | | |
| Jaundice, | 1 | 0 | | | |
| Marasmus, | 0 | 7 | | | |
| Measles, | 0 | 2 | | | |
| Medullary Fungus, | 1 | 0 | | | |
| | — | — | | | |
| *Carried forward,* | 41 | 52 | | | |

Of the above, there were under 1 year, 49

| | | | |
|---|---|---|---|
| From | 1 to 2 | | 29 |
| | 2 to 5 | | 14 |
| | 5 to 10 | | 7 |
| | 10 to 15 | | 2 |
| | 15 to 20 | | 4 |
| | 20 to 30 | | 14 |
| | 30 to 40 | | 13 |
| | 40 to 50 | | 9 |
| | 50 to 60 | | 3 |
| | 60 to 70 | | 7 |
| | 70 to 80 | | 4 |
| | 80 to 90 | | 1 |
| Total, | | | 156 |

Of the above there were 7 from the almshouse, 13 people of colour, and 2 from the country, which are included in the total amount.

*Remarks on the Intermittent and Remittent forms of Fever.* By JNO. F. PETHERBRIDGE, M. D., of Anne Arundel County, Md.—Having had, for some years past, considerable experience in the treatment of the intermittent and remittent forms of fever, and having, with uniform success, pursued a course somewhat different from the one generally adopted, I have thought it might not be altogether uninteresting to the readers of the "Medical and Surgical Journal," to see a brief statement of the plan which I have followed.

In order that the subsequent remarks may be fully appreciated, I would premise that my practising district embraces the lower part of Anne Arundel and the upper part of Calvert counties. A single glance at the map will give a clear idea of its geographical situation. It will be seen to constitute a part of the narrow strip of land running down between the Chesapeake bay on the one side, and the Patuxent river on the other. Thus located, it might be presumed that the situation would be sickly, especially during the autumnal months. And such was the case to a very great degree some ten or fifteen years since. But, owing to the changes which have taken place in the general face of the country, and the alterations which have been effected in the habits of the people, the health of the locality has improved so much, that we do not at this time consider it by any means a sickly one; or at least not near as much so as others similarly exposed.

The diseases with which we have to contend, are those which generally prevail in alluvial situations. For it will be borne in mind that a large portion of our lands is of this character. In the summer, we have diarrhœa, dysentery, and all the modifications of bowel complaints; in the fall, commencing the middle or the last of August, and continuing until the first heavy frost, and for ten days or a fortnight afterwards, we have the whole tribe of diseases which have been generally supposed to owe their origin to malaria, the intermittent, remittent and the well defined congestive fever; and scarcely have these disappeared from amongst us before winter introduces the pneumonia biliosa, a disease which, with us, requires almost as much skill and energy as the congestive fever itself.

But it is, as the caption of our article indicates, upon the intermittent and remittent fever, we propose to offer a few remarks.

Thoroughly educated in what may with propriety be designated the calomel school; taught to believe that the liver is the great source of all the diseases to which mortality is heir, and that calomel is the catholicon by which they are to be overcome, we commenced our professional career, with strong prejudices against every thing that savored of the Broussaic school. But we had been practising but a short time, before we were convinced that however applicable the views we had learned might be to diseases of other climates, they were entirely unsuited to the diseases with which we had to contend. The red tongue, the incessant retching and vomiting, the insatiable thirst, with the tenderness and pain of the epigastric region, so clearly designated the stomach and not the liver as the seat of disease, that the most obtuse intellect could not possibly misunderstand.

But perhaps it is proper to remark here that we have been informed by physicians of great skill and observation, that it has only been since the passing of the cholera through the land, that our fall diseases have assumed a gastric character, that prior to that period, they were entirely different from what they now are, in their pathological indications; and that no form of treatment so rapidly arrested them in their progress, and so successfully relieved them as hydrar submurias in large and repeated doses. So different is the state of things at present, that but for the entire confidence we have in the judgment and the representations of these gentlemen, we could scarcely give credence to an assertion of this character, for we positively aver that in the hundreds of cases which have fallen under our observation, we have never found it necessary in a single instance to resort to this mode of procedure. But viewing them as consisting in gastritis or at most in gastro duodenitis, we have adopted therapeutics in accordance with the same.

If we can see our patient in the early stage of the disease, we invariably bleed during the period of excitement, after which we apply from ten to fifteen cupping glasses over the region of the stomach, duodenum, &c., abstain from all medicines, direct our patient to eat ice "ad libitum," and with the view of extinguishing the excitement as rapidly as possible, we have vessels filled with ice, placed under the hands, so that he may freely play with the same. By these means, we pretty generally succeed in converting what would otherwise be a remittent into an intermittent, and thereby secure to ourselves a more favourable opportunity than we otherwise could have, for the administration of the agent upon which we place our chief reliance in arresting the progress of the disease. It should have been remarked that our remittents almost invariably assume either the simple or the double tertian form; the latter most generally. And if the paroxysmal character of the disease is not overcome, the patient will soon have a dry, chapped tongue, constant fever, great prostration of strength, a low, muttering delirium, and the whole train of symptoms constituting what has been called the typhoid state. To prevent this truly deplorable state, the Materia Medica, in furnishing us with quinine, gives us what approximates as nearly to a specific as it is possible for us to find in the science of therapeutics. But, unfortunately, the intrinsic value of this agent has seldom been fully appreciated, on account of the manner and the quantity in which it has been given. It is true that frequently in the dose of two and three grains every two or three hours, it will suffice in the mild intermittent. But when the disease is one of considerable violence, and where there is danger of the anticipated paroxysm, placing the patient almost if not entirely beyond the reach of the science, such a course is for all the world as was wont to be said by the professor of the theory and practice of medicine in the University of Maryland, like attempting to beat down the rocks of Gibraltar with the grains of mustard seed. Here, if we would rescue our patient, if not from the grave, at least from a long and protracted illness, the remedy must be prescribed in the dose of fifteen or twenty grains. And from years' experience in thus administering it, we hesitate not to affirm that all who will thus prescribe it, will never be induced to abandon the course. This, then, is the plan we pursue, if the disease is one of considerable mildness, and the return of the paroxysm or the exacerbation is not expected for some time after the use of our depletory remedies, we direct a mild laxative. But if the disease is one of violence, and the paroxysm or the excerbation is expected in the course of three or four hours, we never concern ourselves about unloading the bowels, but direct our efforts to the destroying of the periodicity of the disease. And not until after this has been accomplished, do we administer some mild aperient. We are convinced that the routine practice, of purging the patient for days before administering the quinine, is radically wrong. In every instance it keeps up the irritation upon the gastro enteric membrane, and by debilitating the patient, only renders the system more liable to the recurrence of the disease. If the physician can see his patient during the excitement of the first paroxysm, and will freely use his lancet, his cups and ice, and four hours before the expected return will administer the tonic in doses of twenty grains, in a large majority of cases—at least eight out of ten—it will be unnecessary for him to combat with the second. He will have the satisfaction of cutting short, in a few hours, a disease, which, under a different mode of treatment, would require his attention for one, two, three weeks or more, and then have the mortification of seeing his patient with a salivated if not a sloughed mouth.

With propriety the question may be asked, if the administration of so large a dose of quinine is not directly at variance with the pathological views laid down? If the stomach is in a state of inflammation, will not this agent have a direct tendency to increase the same? Reasoning "à priori," would conduct us to such a conclusion. But such, however, is not the case. Vividly is the recollection upon our mind, when we were induced for the first time to make the experiment. The individual had been sick for some time; the case was rapidly passing into the typhoid state; the dry, red, glazed tongue—the certain precursor of this state—was before our eye; and the certainty that the next paroxysm would either result in death, or render the case almost unmanageable, compelled us to make an effort to prevent its return. The tonic was administered, and, to our inexpressible delight, the disease was arrested, and in a short time our patient was convalescent. Since then, we have invariably, after the abstraction of blood, either generally or locally, or both, directed the tonic to be taken; and if we can succeed, as we generally do by the administration of a scruple dose, four hours before the expected paroxysm, in producing the buzzing in the ears—tinnitus aurium—which characterizes the full effect of the agent, we feel perfectly safe. We are assured from repeated observations, that with this sensation, there will be a profuse perspiration, indicating an entire breaking up of the diseased action.

We would remark that we prefer giving the quinine in solution, and instead of using the elixir of vitriol for the purpose of suspending it, we use one of the vegetable acids, generally the tartaric. We dissolve the twenty grains in a table spoonful of water, and add the dry tartaric acid, until the solution becomes transparent. We prefer the vegetable acids as being less irritating in the event of their being slightly in excess; indeed, when we cannot get a distinct intermission for the administration of the tonic, and have to give it during the remission, we prefer making the solution slightly acidulated.

Sometimes it is the case, the paroxysm or the exacerbation comes on an hour or two sooner than was anticipated, and not being able to foresee this, the quinine has not been administered sufficiently early to produce its specific influence in time, in this case the second paroxysm is developed; but by guarding against it at the next period, by giving the tonic sufficiently early to produce the tinnitus aurium, we at once put a stop to it. As before stated, from three to four hours is the period we prefer.

Briefly, and in a desultory manner, such is the course we pursue in the treatment of these diseases; and we have the satisfaction of knowing that of some hundreds of cases including the intermittent, remittent, and the congestive forms, we have never lost but a single case of bilious fever.

In conclusion, we propose to subjoin one or two cases illustrative of our views. Numbers might be added, but as the treatment and the results were precisely the same, it is unnecessary to extend our paper.

*Case* 1.—Sept. 21. Called to-day to see my estimable friend, Dr. Hammond Stewart. He informs me that he has been sick for nearly a week; at present, he is in the cold stage of the disease; suffers excruciatingly from the chill; violent pain in the back of the head; retching and vomiting, with insatiable thirst; after waiting for an hour, the reaction developed itself; bled him freely from the arm; directed ice to be used ad libitum; absolute diet, and abstinence from all medicines. Anticipating a return at ten or eleven o'clock the next day, we left from twenty to twenty-five grains of quinine, with directions for it to be administered at one dose.

Sept. 22. Visited him at half past ten, A. M. The fever had continued nearly all night, notwithstanding the bleeding and the ice, but at six in the morning, having an apyrexial period, took the tonic; find him free from fever, and in a profuse perspiration from head to foot. This is an effect which I have almost invariably seen to follow large doses of quinine after depleting the system. He sensibly experiences the roaring in his ears. We feel confident from his present state that he will have no return.

Sept. 23. No return of the paroxysm; bowels being constipated, prescribed a laxative; on account of the smallness of its bulk, he preferred a blue pill.

Sept. 24. Found him riding out upon his farm.

*Case* 2.—Sept. 18. Called in consultation to visit Mrs. W———, ætat. from fifty to sixty. Informed by her physician that she had been sick for nearly a week. Her disease was a double tertain. Calomel and the agents generally employed, had been freely used, but without avail. She was becoming more and more prostrated; tongue red and dry; great thirst. It is now eleven o'clock, A. M.; at two P. M., she expects a return of the paroxysm. She is entirely too weak to lose blood from the general system. Cupped her over the epigastrium; applied an epispastic, and administered from fifteen to twenty grains of quinine dissolved as before stated. At one P. M., she began to hear the buzzing in her ears, perspiring freely throughout the whole system.

Four, P. M. Missed her paroxysm; confident from the specific influence of the agent having been produced, that there will be no return unless she exposes herself, we took our leave, not intending to return. A few days afterwards, we saw her physician, who informed us that she had so far recovered as to render his visiting unnecessary.

In both of the above cases, the quinine was administered during an intermission; but the following will show the propriety of the course, not only when an intermission cannot be obtained, but also under circumstances which would appear positively to contra-indicate it.

*Case* 3.—August —. Called to see Miss. S. K———, ætat. about nineteen; a slender form and bilious temperament. Having some time previously exposed herself to the vicissitudes of the weather, she had contracted what is commonly designated a bad cold, attended with a violent pain in the breast and a distressing cough. A few days since, she was attacked with a severe chill, ever since which she has been suffering from a burning fever; complains of violent pain in the back, head, and breast. The slightest pressure upon the epigastrium produces pain; the pulse is full, strong, and frequent; with the exception of a slight glaze, the tongue with propriety might be compared to raw beef. Her thirst is so great, no quantity of ice will satisfy her. Bled about twenty ounces from the arm; cupped freely over the stomach, duodenum, and breast, directed the free use of ice both externally and internally. Oleum Ricini ℥j. Upon visiting her the next morning, found that upon the whole she had passed a comfortable night, but still a great degree of excitement in the system. Unwilling to administer the tonic while the fever continued, directed the continued use of ice, hoping to be able to subdue the same; left from fifteen to twenty grains of quinine to be given in the event of any thing approximating to an intermission. But upon visiting her the following morning, was informed, that shortly after I had left, the fever had increased, and had continued to rage until a few hours previously, when it appeared to abate a little. She was evidently succumbing under the force of the disease; and as the presumption was that she was no freer from fever than she would be during the day, I determined to delay no longer. The quinine was given; no exacerbation came on during the day. The next day she was nearly if not entirely free from fever. From this time she began to amend. The pulmonic symptoms gradually subsided, and, with the exception of an occasional laxative, no other medicines were used. Perhaps some may be disposed to doubt the propriety of administering the tonic in a case so violent and so complicated as the above. The result proved the correctness of the course; and it is our decided opinion that under any other treatment the case must have terminated fatally. We close by giving it as the opinion which we have deduced from multiplied observations, that quinine is not the stimulant that it has been generally supposed, and that in the remission of fevers, signal benefit will result from its exhibition in large doses, if the system has been prepared for its reception.—*Maryland Med. and Surg. Journ.*

